# Associations between fatty liver index and asymptomatic intracranial vertebrobasilar stenosis in Chinese population

**DOI:** 10.1371/journal.pone.0187502

**Published:** 2017-11-09

**Authors:** Jing Qiu, Zhiwei Zhou, Jie Wang, Yiying Wang, Chunxing Liu, Xingxiang Liu, Yunfang Xu, Lugang Yu, Hui Zhou, Jie Lin, Zhirong Guo, Chen Dong

**Affiliations:** 1 Department of Epidemiology and Statistics, School of Public Health, Jiangsu Key Laboratory and Translational Medicine for Geriatric Disease, Medical College of Soochow University, Suzhou, China; 2 Department of Laboratory Medicine, East China Sanatorium, Wuxi, China; 3 Department of Laboratory Medicine, Huai’an Forth Hospital, Huai’an, China; 4 Suzhou Industrial Park Centers for Disease Control and Prevention, Suzhou, China; 5 Suzhou Vocational Health College, Suzhou, China; Medizinische Fakultat der RWTH Aachen, GERMANY

## Abstract

Metabolic diseases such as type 2 diabetes mellitus (T2DM) and metabolic syndromes (MetS) have been recognized as the important risk factors for asymptomatic intracranial vertebrobasilar stenosis (IVBS). Although fatty liver index (FLI) is significantly related with these diseases, the association between FLI and IVBS remains unclear. In the present study, 2368 participants (30–75 years) were recruited from a Chinese prospective cohort study of PMMJS. Amongst them, 2281 individuals who did not have IVBS at baseline were enrolled in the 6-year following-up study. In cross-sectional analysis based on the baseline characteristics, the results showed that FLI was positively related with IVBS prevalence. Compared to the participants with FLI < 30, the adjusted OR (95% CI) of IVBS was 2.07 (1.18, 3.62) and 2.85 (1.39, 5.18) in the groups of 30 ≤ FLI < 60 and FLI ≥ 60, respectively. In longitudinal analysis, the results showed that the participants with FLI ≥ 60 had an increased risk of asymptomatic IVBS compared to those with FLI < 30 [adjusted HR (95%CI): 1.65 (1.05, 2.60)]. Moreover, exclusion of persons with hypertension, T2DM and MetS did not alter the associations between FLI and asymptomatic IVBS. Therefore, our results suggest that elevated FLI is an independent risk factor for asymptomatic IVBS in Chinese adults.

## Introduction

Vertebrobasilar system is comprised of the vertebral arteries and the basilar artery. Each vertebral artery arises from a branch of the subclavian artery, and merges at the pontomedullary junction to form the basilar artery. Intracranial vertebrobasilar stenosis (IVBS) is a common cause of ischemic stroke and an important risk factor of stroke recurrence [1, 2]. Clinically, up to 20% of ischemic stroke is in the vertebrobasilar circulation territory [3, 4]. Therefore, it is critical to analyze the risk factors of asymptomatic IVBS and their effects on the disease prognosis in stroke patients.

Over the last two decades, non-alcoholic fatty liver disease (NAFLD) has emerged as the leading cause of chronic liver disease worldwide [5]. The disease is associated with clinical states such as obesity, insulin resistance, and type 2 diabetes mellitus (T2DM), and covers a wide range of liver changes, ranging from simple steatosis to non-alcoholic steatohepatitis (NASH), liver cirrhosis, and hepatocellular carcinoma [6–8]. Further to NAFLD’s substantial effects on liver physiology, more and more studies have reported independent associations between NAFLD and major cardiovascular events [9–11].

Fatty liver index (FLI) is a noninvasive index for the prediction of hepatic fatty liver based on body mass index (BMI), triglyceride (TG), gamma-glutamyltransferase (GGT) and waist circumference (WC) [12]. In a Chinese study including 8626 adolescents, Huang et al. reported that NAFLD determined by FLI was well correlated with the hepatic steatosis diagnosed by abdominal ultrasonography. The optimal cut-off point of the FLI for diagnosing NAFLD was 30 with a high sensitivity of 79.89% and a specificity of 71.51% [13]. Moreover, the results from RISC study have shown that subjects with more than 60 of FLI value were at higher risk of atherosclerotic lesions, independently of traditional factors such as the components of MetS [14]. Given that atherosclerosis is an important pathogenic mechanism related with IVBS development [15], we therefore conducted the present study to investigate the associations between FLI and asymptomatic IVBS based on the Chinese cohort study of “The prevention of MS and multi-metabolic disorders in Jiangsu” (PMMJS).

## Materials and methods

### Study population

**Fig 1:**
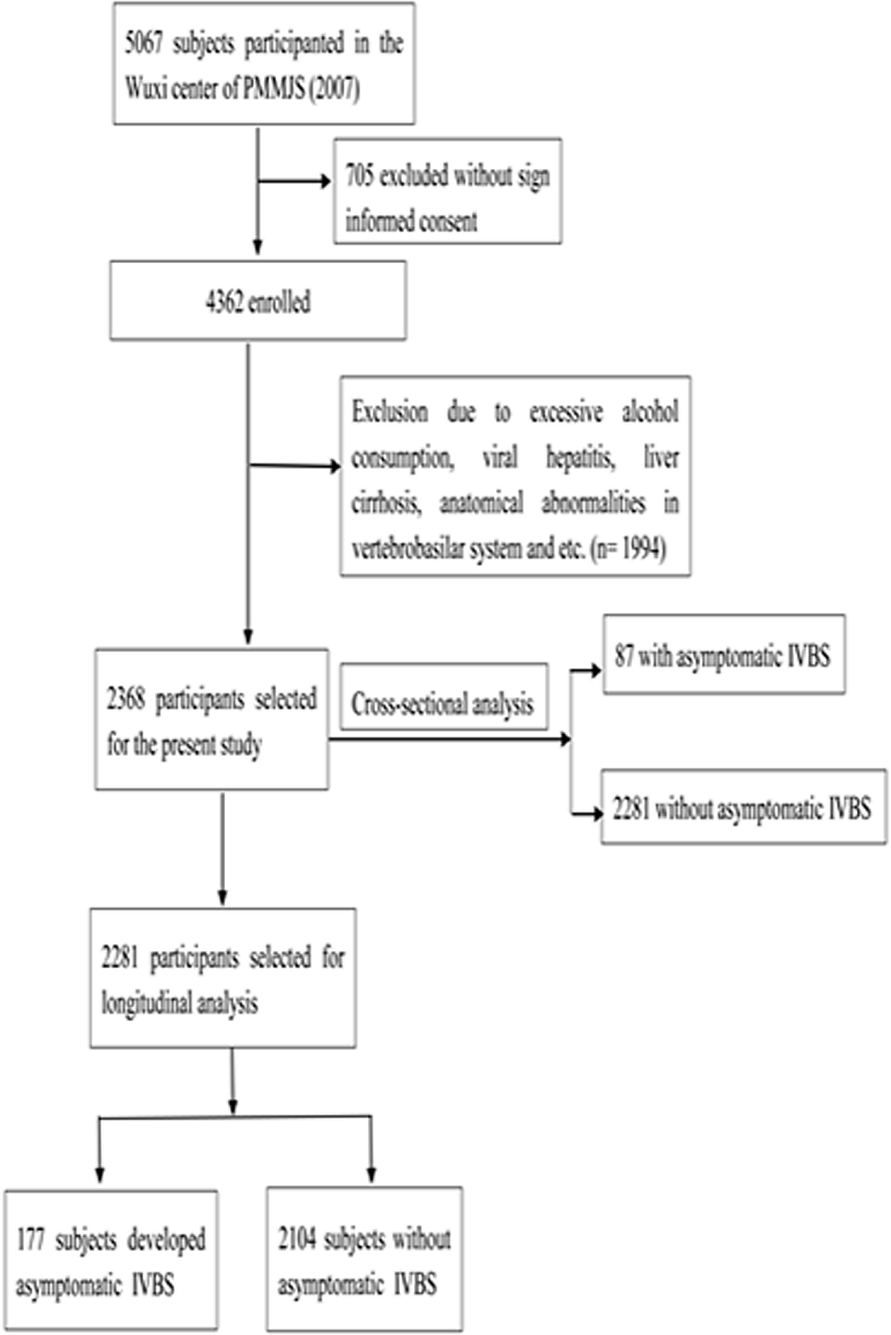
Study flow diagram for the included participants.

The subjects included in the present study were recruited from the Wuxi center of PMMJS, which was established at early 2000 and aimed to analyze the epidemiologic characteristics of chronic diseases in Jiangsu, China. The design, protocol and sampling method of PMMJS have been described in detail before [16]. In short, 5067 subjects (aged 30–75 years) were enrolled in PMMJS study at baseline. Amongst them, 4362 individuals accepted the invitation to participate in this study between February and August in 2007. We excluded the subjects with excessive alcohol consumption (≥ 30 g/day for men or ≥ 20 g/day for women) (874), liver cirrhosis or suspicion of malignancy (39), HBsAg positive (355) or anti-HCV antibody positive (10) and a history of schisomiasis (29). Additionally, 23 persons with anatomical abnormalities in vertebrobasilar system, 291 hypertensive individuals under anti-hypertension treatment, 58 T2DM patients under glucose-lowing treatment, and 315 participants under lipid-lowering treatment were excluded. At last, 2368 subjects at baseline who completed a large scale of physical examination were included for the cross-sectional analysis. Meanwhile, after excluding 87 individuals with asymptomatic IVBS at baseline, 2281 subjects were enrolled in a 6-year follow-up study from 2007 to 2013 (Fig 1).

This work was approved by the ethical committee of Soochow University and was conducted in accordance with the ethical standards of the responsible committees for human experimentation and with the Helsinki Declaration of 1975. All of participants were asked to provide written informed consent before data collection.

### Anthropometric measurements and data collection

The demographic information (age, gender, weight and height), lifesyle risk factors (cigarette smoking and alcohol drinking), history of disease (CVD, T2DM, hypertension and intracranial arterial stenosis) of participants were collected. Height was accurate to 0.1 cm and weight was measured within 0.1 kg. BMI was calculated as the ratio of weight in kilograms to height in meters squared. Three consecutive sitting blood pressure measurements (one minute between each) were taken by trained staff using a mercury sphygmomanometer with a standard protocol, after the subjects had been resting for about 5 minutes.

### Laboratory measurements

An overnight fasting blood sample was collected from each participant. The fasting plasma glucose (FPG), TG, total cholesterol (TC), low density lipoprotein cholesterol (LDL-c), high density lipoprotein cholesterol (HDL-c), alanine aminotransferase (ALT), aspartate aminotransferase (AST) and GGT were measured with standard laboratory procedures by using an autoanalyzer (Olypums AU640, Japan). The intra-coefficients of variation values were less than 5.0% and the inter-assay values were less than 9.5% for all examinations.

### Fatty liver index

Based on the following formula: FLI = (e^0.953^×log_e_[TG]+0.139×BMI+0.718×log_e_[GGT]+0.053×WC-15.745)/(1+e^0.953^×log_e_[TG]+0.139×BMI+0.718×log_e_[GGT]+0.053×WC-15.745) ×100. The units of TG, GGT, and WC were mmol/L, U/L, and cm, respectively. In the present study, we categorized the study participants into 3 groups based on the value of FLI which ranges 0 to 100. Group 1: FLI<30, described as low FLI; Group 2: 30 ≤ FLI < 60, defined as intermediate FLI; Group 3: FLI ≥ 60, defined as high FLI.

### TCD examination

TCD is widely used in epidemiological studies for the diagnosis of intracranial arterial stenosis because it is a noninvasive, effective and reliable method [17, 18]. In the present study, TCD examination was performed by two experienced radiologist using a portable devices (EME Companion, Nicolet). Both neurologists when performing the sonography were unaware of the baseline information of the participants. A diagnosis of IVBS was established following peak systolic flow velocity (PSV) criteria: > 100 cm/second for the vertebral or basilar arteries, which have been published and validated against MR angiography elsewhere [19, 20]. For participants over 60 years old, the criteria would be lower by 20 cm/s. In addition to the PSV criteria, the participants’ age, disturbance of the echo frequency, turbulence or abnormal blood flow were also considered for the diagnosis [19]. Asymptomatic IVBS was diagnosed if at least one of the studied arteries showed evidence of stenosis. Undetected arteries via both temporal and orbital window were considered negative [19].

### Definition of hypertension, T2DM and MetS

The definition of hypertension was: (1) history of hypertension diagnosed by a physician; (2) SBP ≥ 140 mmHg, and/or DBP ≥ 90 mmHg; or (3) currently underwent antihypertensive treatment prescribed by a physician. T2DM was defined as: (1) FPG levels were ≥ 7.0 mmol/L; or (2) history of T2DM which was diagnosed by a physician; (3) individual was undergoing treatment with insulin or oral hypoglycemic agents. MetS was diagnosed when the participants had 3 or more of the following components: (1) WC ≥ 90 cm for men and ≥ 80 cm for women; (2) TG ≥ 1.7 mmol/l; (3) HDL-c < 1.03 mmol/l; (4) SBP ≥ 130mmHg and/or DBP ≥ 85mmHg or with previously diagnosis of hypertension and current use of antihypertension medications; and (5) FPG ≥ 5.6 mmol/l or previous diagnosis with T2DM.

### Statistical analysis

Statistical analyses were performed using SAS 9.4 software (SAS Institute, Cary, NC, USA). Data were expressed as means ± SD or frequencies. The unpaired t-test and the χ^2^-test with Yates’s correction for continuity (for categorical variables) were used to compare the characteristics of participants. In cross-sectional analysis, all of participants were divided into two groups according to the participants with and without IVBS at baseline. Multivariate logistic regression analysis was performed to assess the independent association of baseline FLI with the prevalence of asymptomatic IVBS. The odds ratios (OR) and 95% confidence intervals (CIs) were analyzed. In longitudinal analysis, the cox regression was used to estimate the associations of FLI with incidence of asymptomatic IVBS over 6 years, and crude and adjusted hazards ratios (HR) and their 95% CI were calculated. In the fully adjusted regression model, the variables included gender (male/female), age, BMI, smoking status (yes/no), SBP, DBP, HDL-c, LDL-c, TC, FPG, ALT and AST. All reported *P* values were two-tailed, and those < 0.05 were considered statistically significant.

## Results

### Cross-sectional associations between FLI and asymptomatic IVBS

The characteristics of 2368 participants with or without IVBS (87 *vs* 2281) were presented in Table 1. As the results shown, the mean FLI value of the subjects with IVBS was 57.20 ± 6.66, which was significantly higher than those without IVBS (50.89 ± 9.82, P = 0.000). As expected, the prevalence of hypertension, T2DM and MetS was much higher in individuals with IVBS. Moreover, participants with IVBS were older and had higher BMI, WC, FPG, TG and lower HDL-c compared to those without IVBS. However, no significant differences were observed in ALT, AST and GGT between two groups.

**Table 1:** Characteristics of participants with or without IVBS in cross-sectional analysis.

Characteristics	No asymptomatic IVBS (n = 2281)	asymptomatic IVBS (n = 87)	P Value
Age (years)	50.89±9.82	57.20±6.66	0.000
Men (%)	1837(80.53%)	83(95.40%)	0.001
Smoking (%)	954(41.82%)	42(48.28%)	0.232
BMI (kg/m^2^)	23.65±2.97	25.71±2.63	0.000
WC (cm)	82.28±8.59	88.86±6.63	0.000
SBP (mmHg)	116.2±12.84	120.5±14.00	0.002
DBP (mmHg)	75.77±9.10	80.02±9.63	0.000
ALT (U/L)	25.86±17.30	29.02±19.93	0.092
AST (U/L)	22.89±7.53	24.46±9.19	0.068
GGT (U/L)	33.54±34.04	36.47±23.74	0.199
FLI	28.88±23.34	44.63±24.55	0.000
FPG (mmol/L)	5.40±0.85	5.71±0.82	0.001
TG (mmol/L)	1.63±1.42	2.05±1.69	0.026
TC (mmol/L)	4.72±0.83	4.83±0.75	0.246
LDL-c (mmol/L)	2.89±0.73	2.95±0.70	0.432
HDL-c (mmol/L)	1.30±0.31	1.19±0.30	0.002
Hypertension (%)	214(9.38%)	20(22.99%)	0.000
T2DM (%)	57(2.50%)	6(6.90%)	0.012
MetS (%)	68(2.98%)	8(9.20%)	0.001

BMI: body mass index; WC: waist circumference; SBP: systolic blood pressure; DBP: diastolic blood pressure; ALT: alanine aminotransferase; AST: aspartate aminotransferase; GGT: gamma-glutamyltransferase; FLI: fatty liver index; FPG: fasting plasma glucose; TG: triglycerides; TC: total cholesterol; LDL-c: low density lipoprotein cholesterol; HDL-c: high-density lipoprotein cholesterol; T2DM: type 2 diabetes mellitus; MetS: metabolic syndromes.

The overall prevalence of IVBS was 1.95%, 5.28% and 8.21% in the groups of FLI < 30, 30 ≤ FLI < 60 and FLI ≥ 60, respectively. Compared to the participants with FLI < 30, the OR (95% CI) of IVBS was 2.07 (1.18, 3.62) and 2.68 (1.39, 5.18) in the groups of 30 ≤ FLI < 60 and FLI ≥ 60 respectively, after adjusting for confounding variables (Table 2). Furthermore, we performed the sensitivity analysis to evaluate the cross-sectional associations between FLI and IVBS. The results showed that exclusion of participants with hypertension, T2DM and MetS did not alter the association between FLI and prevalence of asymptomatic IVBS (OR FLI ≥ 30 vs FLI < 30: 2.44, 95% CI: 1.26–4.74) (Table 3).

**Table 2:** Associations of FLI with IVBS in cross-sectional analysis.

	FLI<30 (n = 1433)	30≤FLI<60 (n = 606)	FLI≥60 (n = 329)	*P*
IVBS cases	28	32	27	
Crude OR	1.00(ref)	2.80(1.67,4.69)	4.49(2.61,7.72)	<0.0001
Model 1	1.00(ref)	2.06(1.21,3.53)	2.92(1.64,5.21)	0.0002
Model 2	1.00(ref)	2.07(1.18,3.62)	2.68(1.39,5.18)	0.0026

Model 1: Adjusted for age, gender, BMI, smoking, SBP and DBP

Model 2: Model 1 and ALT, AST, HDL-c, LDL-c and FPG.

**Table 3:** Sensitivity analysis on the association between FLI with IVBS in cross-sectional analysis.

	Cases (%)	Unadjusted		Model 1		Model 2	
		OR (95%CI)	*P*	OR (95%CI)	*P*	OR (95%CI)	*P*
FLI < 30 (n = 1287)	23 (1.79%)	1.00 (ref)	—	1.00 (ref)	—	1.00(ref)	—
FLI ≥30 (n = 462)	26 (5.63%)	3.28 (1.85,5.80)	0.000	2.69 (1.46,5.96)	0.002	2.44 (1.26,4.74)	0.008

Model 1: Adjusted for age, gender, BMI, smoking, SBP and DBP

Model 2: Model 1 and ALT, AST, HDL-c, LDL-c and FPG.

### Longitudinal association between FLI and asymptomatic IVBS

Totally, 177/2281 (7.76%) persons were diagnosed with asymptomatic IVBS during six years follow-up. However, we did not observe stroke cases in the present longitudinal study. Anthropometric, clinical, and laboratory data of studied persons were shown in Table 4. Compared to the persons without asymptomatic IVBS, the subjects with asymptomatic IVBS were about 4.6 years older, and had higher serum levels of GGT, TG, TC and FPG. As expected, HDL-c levels were significantly lower in individuals with asymptomatic IVBS.

**Table 4:** Baseline characteristics of participants with or without asymptomatic IVBS in longitudinal analysis.

Characteristics	No asymptomatic IVBS (n = 2104)	asymptomatic IVBS (n = 177)	*P*
Age (years)	50.53±9.92	55.14±7.30	0.000
Men (%)	1667(79.23%)	170(96.05%)	0.000
Smoking (%)	877(41.68%)	77(43.50%)	0.637
BMI (kg/m^2^)	23.52±2.81	25.18±4.16	0.000
WC (cm)	81.92±8.63	86.49±6.89	0.000
SBP (mmHg)	115.8±12.73	120.6±13.37	0.000
DBP (mmHg)	75.42±8.94	79.86±9.89	0.000
ALT (U/L)	25.58±17.26	28.94±17.45	0.004
AST (U/L)	22.79±7.58	23.95±6.98	0.022
GGT (U/L)	32.83±32.59	41.22±46.36	0.006
FLI	27.94±22.98	40.04±24.70	0.000
FPG (mmol/L)	5.39±0.85	5.58±0.82	0.004
TG (mmol/L)	1.60±1.41	1.96±1.55	0.001
TC (mmol/L)	4.71±0.82	4.87±0.89	0.013
LDL-c (mmol/L)	2.88±0.73	2.99±0.73	0.050
HDL-c (mmol/L)	1.30±0.31	1.22±0.27	0.000
Hypertension (%)	457(17.52%)	73(30.17%)	0.000
T2DM (%)	129(4.94%)	14(5.79%)	0.567
MetS (%)	152(5.83%)	32(13.22)	0.000

BMI: body mass index; WC: waist circumference; SBP: systolic blood pressure; DBP: diastolic blood pressure; ALT: alanine aminotransferase; AST: aspartate aminotransferase; GGT: gamma-glutamyltransferase; FLI: fatty liver index; FPG: fasting plasma glucose; TG: triglycerides; TC: total cholesterol; LDL-c: low density lipoprotein cholesterol; HDL-c: high-density lipoprotein cholesterol; T2DM: type 2 diabetes mellitus; MetS: metabolic syndromes.

**Fig 2:**
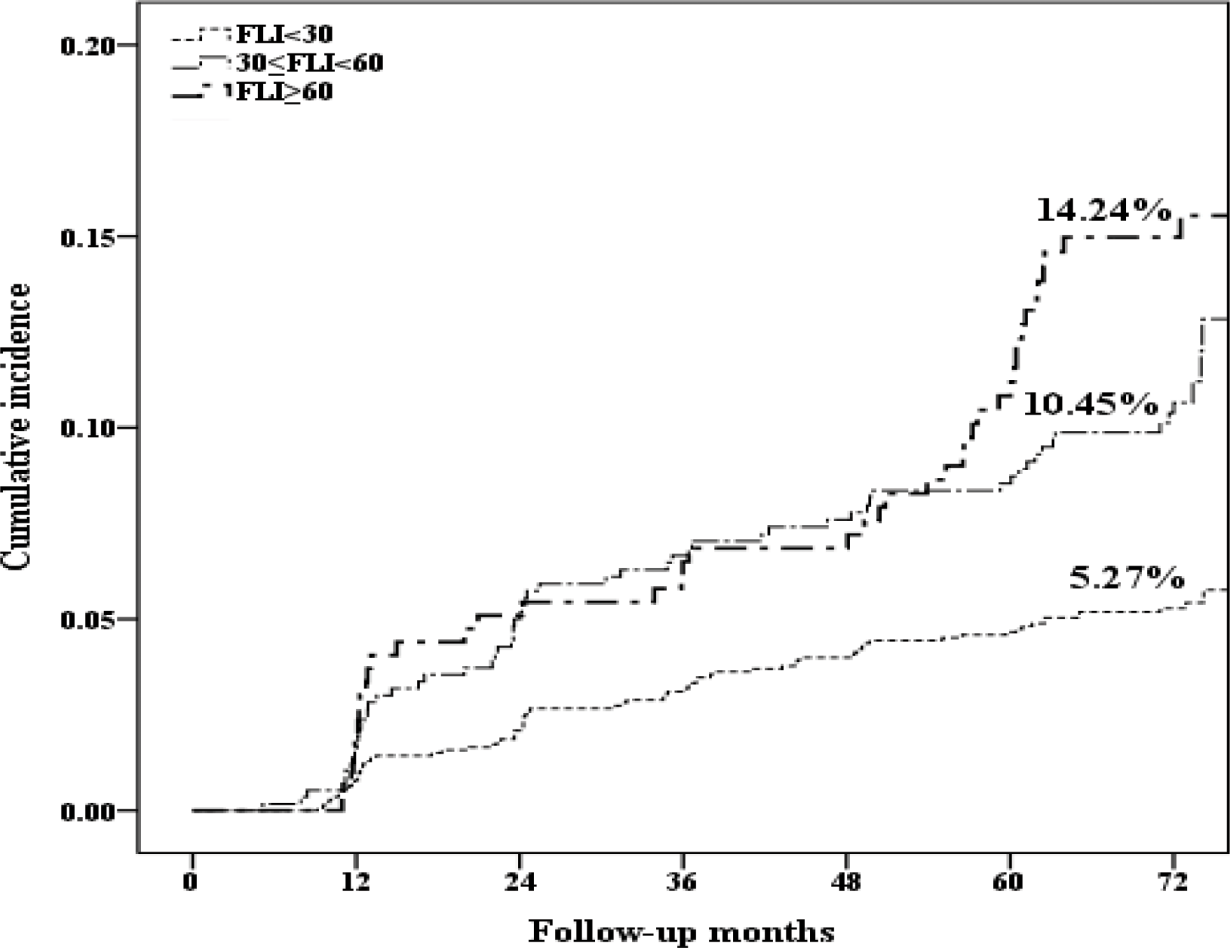
The cumulative incident asymptomatic IVBS in three groups. The results of Kaplan–Meier analysis showed that there was a significantly higher incidence of asymptomatic IVBS in participants with higher FLI compared to those with FLI < 30 (P < 0.001 for both FLI < 30 vs 30 ≤ FLI < 60 and vs FLI ≥ 60, respectively).

As shown in Fig 2, the results of Kaplan–Meier analysis showed that there was an increased incidence of asymptomatic IVBS in participants with intermediate and high FLI values. Compared to the subjects in the FLI < 30 group, subjects in 30 ≤ FLI < 60 and FLI ≥ 60 groups had HR (95% CI) of 2.04 (1.45, 2.87) and 2.80 (1.92, 4.07) for the unadjusted model, respectively. Moreover, the individuals with FLI ≥ 60 had a 1.65 fold increased risk of asymptomatic IVBS compared to those with FLI < 30, even after adjusting for confounding variables (Table 5).

**Table 5:** Associations of FLI with IVBS in cross-sectional analysis.

	FLI<30 (n = 1405)	30≤FLI<60 (n = 574)	FLI≥60 (n = 302)	*P*
IVBS cases	74	60	43	
Crude HR	1.00 (ref)	2.04 (1.45, 2.87)	2.80 (1.92, 4.07)	0.000
Model 1	1.00 (ref)	1.39 (0.98, 1.98)	1.71 (1.15, 2.54)	0.006
Model 2	1.00 (ref)	1.38 (0.97, 1.97)	1.65 (1.05, 2.60)	0.025

Model 1: Adjusted for age, gender, BMI, smoking, SBP and DBP

Model 2: Model 1 and ALT, AST, HDL-c, LDL-c and FPG.

Table 6 and S1 Table showed subgroup analysis for incident asymptomatic IVBS of the participants with FLI ≥ 30 using FLI <30 as the reference. The results showed that in the group of FLI ≥30, the participants with less than 51 years, higher levels of GGT, and lower level of TC, LDL-c, HDL-c and FPG, had an increased risk of the incidence of asymptomatic IVBS. However, we did not observe the significant effects of the serum ALT and AST levels on the association between FLI and the incident asymptomatic IVBS.

**Table 6:** Stratified analysis on the association between FLI and asymptomatic IVBS according to ALT, AST and GGT in longitudinal study (FLI < 30 *vs* FLI ≥ 30).

	Unadjusted		Adjusted	
	HR (95% CI)	*P*	HR (95% CI)	*P*
ALT (U/L)				
<21	2.28 (1.34,3.89)	0.002	1.21 (0.67,2.18)	0.520
≥21	1.85 (1.23,2.78)	0.003	1.52 (0.98,2.37)	0.064
AST (U/L)				
<21	2.74 (1.65,4.54)	0.000	1.49 (0.83,2.66)	0.183
≥21	1.87 (1.28,2.75)	0.001	1.32 (0.85,2.06)	0.215
GGT (U/L)				
<22	2.06 (1.07,3.97)	0.031	0.99 (0.48,2.06)	0.982

Adjusted for age, gender, BMI, smoking, SBP, DBP, HDL-c, LDL-c and FPG. When compared in different groups, HR (95% CI) was calculated by adjustment for other liver enzymes.

As shown in Table 7, almost identical results were obtained in further sensitivity analysis. After excluding the subjects with hypertension, T2DM and MetS, the result still showed that participants with FLI ≥ 30 had an increased risk of IVBS compared to those with FLI less than 30, after adjusting for confounding variables (HR: 1.44, 95% CI: 1.02–2.04).

**Table 7:** Sensitivity analysis on the association between FLI with IVBS in cross-sectional analysis.

	Cases (%)	Unadjusted		Model 1		Model 2	
		HR (95%CI)	*P*	HR (95%CI)	*P*	HR (95%CI)	*P*
FLI < 30 (n = 1287)	63 (4.98%)	1.00 (ref)	—	1.00 (ref)	—	1.00(ref)	—
FLI ≥30 (n = 462)	49 (11.24%)	2.33 (1.60,3.38)	0.000	1.61 (1.09,2.37)	0.017	1.44 (1.02,2.04)	0.038

Model 1: Adjusted for age, gender, BMI, smoking, SBP and DBP

Model 2: Model 1 and ALT, AST, HDL-c, LDL-c and FPG.

## Discussion

This study has shown that FLI has a cross-sectional association with prevalent asymptomatic IVBS and a prospective association with incident asymptomatic IVBS in Chinese adults. The participant with elevated FLI value has an increased risk of asymptomatic IVBS development. Furthermore, this association remains statistically significant even after excluding the subjects with traditional risks such as hypertension, T2DM, MetS and etc.

A large number of studies from Chinese, Korean and European reported that FLI was well correlated with the NAFLD diagnosed by abdominal ultrasonography, although FLI was originally proposed to identify those individuals who should be submitted to liver ultrasonography for suspected fatty liver [12–14, 21–23]. In a recent Italian study, Targher et al. suggested that NAFLD was associated with a remarkably higher prevalence of asymptomatic/symptomatic coronary, cerebrovascular or peripheral vascular disease. This association remained statistically significant even after adjustment for several established risk factors, including the components of the metabolic syndrome [24]. Additionally, in a pooled analysis of 19,274 adult participants from 20 studies, the results showed that participants with NAFLD had an ~ 18.70% increase in carotid intimal medial thickness when compared with those without NAFLD. Moreover, NAFLD was found to be significantly related with an increased risk of carotid plaque development in 14,445 adult participants from 13 studies [25]. These previous findings might partially explain the relationships between FLI and asymptomatic IVBS.

ALT, AST and GGT are widely used liver tests [26]. Recently, a number of studies reported that raised serum activity of ALT, AST and GGT independently predicted the future development of MetS and T2DM as well as cardiovascular events and/or total/CVD mortality [27–31]. Consistent with these findings, our present study found that the risk of asymptomatic IVBS increased with FLI values in the participants with higher levels of GGT, but not in the subjects with lower serum GGT levels. Therefore, the present study suggests that those NAFLD individuals exhibiting raised liver enzymes may have a higher risk of IVBS.

In stratified analysis, we observed that individuals with FLI ≥ 30 had an increased risk of incident IVBS in the subgroup with lower levels of FPG, TC, TG, HDL-c and LDL-c. NAFLD patients exhibit an atherogenic dyslipidemia that is generally characterized by an increased plasma concentration of TG, reduced concentration of HDL-c, and LDL particles that are smaller and more dense than normal [32, 33]. Therefore, a better understanding of features and mechanisms of atherogenic dyslipidemia occurring in NAFLD individuals is key and further studies should be addressed at better characterizing it.

Our study has several limitations. First, although TCD has been regarded as a reliable screening method to diagnose IVBS according to the Stroke Outcomes and Neuroimaging of Intracranial Atherosclerosis (SONIA) trial [34, 35], it is not as accurate as DSA for IVBS determination. Second, the presence of IVBS was classified as yes or no, instead of degree of stenosis in the present study; thus the potential associations between FLI and the severity of IVBS cannot be tested further. Lastly, our study population was not a nationally representative sample and the findings may not be applicable to other ethnic and regional populations.

In summary, our present study provided the evidence that persons with elevated FLI have higher risks of asymptomatic IVBS and the likelihood of asymptomatic IVBS increased with FLI indices. In future, more prospective studies are needed to establish whether FLI is associated with the progression of asymptomatic IVBS and its related diseases in different population.

## Supporting information

S1 Table Stratified analysis on the associations between FLI and asymptomatic IVBS in longitudinal study (FLI < 30 vs FLI ≥ 30). When compared in different groups, HR (95% CI) was calculated by adjustment for other covariates.
(DOCX)

S2 Table The original information of studied population for cross-sectional analysis. (XLSX)

S3 Table The original information of studied population for longitudinal analysis. (XLSX)
