# Supplementary material for: Associations between fatty liver index and asymptomatic intracranial vertebrobasilar stenosis in Chinese population
Source: PLoS One. 2017 Nov 9;12(11):e0187502. doi: 10.1371/journal.pone.0187502 (PMC5679613; doi:10.1371/journal.pone.0187502)
Supplement: S1 Table — When compared in different groups, HR (95% CI) was calculated by adjustment for other covariates. (DOCX) [file pone.0187502.s001.docx]

Supplement Table 1 Stratified analysis on the association between FLI and asymptomatic IVBS in longitudinal study (FLI<30 *vs* FLI≥30)

|  |  | Unadjusted | |  | Adjusted | |
| --- | --- | --- | --- | --- | --- | --- |
|  |  | OR (95%CI) | P |  | OR (95%CI) | P |
| Age (years) | <51 | 4.09 (2.22,7.53) | 0.000 |  | 2.91 (1.42,5.98) | 0.004 |
|  | ≥51 | 1.62 (1.15,2.28) | 0.006 |  | 1.15 (0.77,1.71) | 0.498 |
| Gender | Males | 1.74 (1.29,2.37) | 0.000 |  | 1.36 (0.96,1.93) | 0.085 |
|  | Females | - | - |  | - | - |
| Smoke | Yes | 1.66 (1.06,2.61) | 0.028 |  | 1.27 (0.75,2.16) | 0.369 |
|  | No | 2.95 (1.98,4.39) | 0.000 |  | 1.58 (0.99,2.52) | 0.055 |
| BMI (kg/m^2^) | <24 | 1.76 (0.88,3.49) | 0.108 |  | 1.06 (0.50,2.27) | 0.875 |
|  | ≥24 | 1.70 (1.12,2.56) | 0.012 |  | 1.34 (0.85,2.10) | 0.205 |
| SBP (mm/Hg) | <120 | 2.90 (1.76,4.77) | 0.000 |  | 1.78 (0.97,3.28) | 0.062 |
|  | ≥120 | 1.73 (1.20,2.53) | 0.004 |  | 1.20 (0.78,1.84) | 0.410 |
| DBP (mm/Hg) | <75 | 2.42 (1.44,4.07) | 0.001 |  | 1.56 (0.85,2.88) | 0.151 |
|  | ≥75 | 1.87 (1.28,2.72) | 0.001 |  | 1.49 (0.97,2.27) | 0.067 |
| FBG (mmol/L) | <5.31 | 2.57 (1.64,4.13) | 0.000 |  | 1.79 (1.04,3.07) | 0.035 |
|  | ≥5.31 | 1.90 (1.28,2.83) | 0.002 |  | 1.24 (0.78,1.95) | 0.362 |
| TC (mmol/L) | <4.70 | 2.66 (1.70,4.17) | 0.000 |  | 1.71 (1.01,2.91) | 0.046 |
|  | ≥4.70 | 1.94 (1.30,2.91) | 0.001 |  | 1.25 (0.78,2.01) | 0.349 |
| TG (mmol/L) | <1.36 | 2.52 (1.40,4.53) | 0.002 |  | 1.42 (0.73,2.74) | 0.303 |
|  | ≥1.36 | 1.68 (1.11,2.54) | 0.015 |  | 1.24 (0.79,1.94) | 0.358 |
| HDL-c (mmol/L) | <1.22 | 2.54 (1.46,3.47) | 0.000 |  | 1.73 (1.09,2.77) | 0.021 |
|  | ≥1.22 | 2.06 (1.27,3.35) | 0.004 |  | 1.19 (0.69,2.04) | 0.532 |
| LDL-c (mmol/L) | <2.91 | 3.76 (2.36,5.99) | 0.000 |  | 2.14 (1.23,3.72) | 0.007 |
|  | ≥2.91 | 1.51 (1.02,2.24) | 0.038 |  | 1.08 (0.69,1.69) | 0.747 |

When compared in different groups, HR (95% CI) was calculated by adjustment for other covariates.
